# Environmental context shapes sex-specific costs of reproduction in a dioecious plant

**DOI:** 10.1093/aob/mcaf296

**Published:** 2025-11-14

**Authors:** Matthew T Gibson, R J Neil Emery, Marcel E Dorken

**Affiliations:** Environmental and Life Sciences Graduate Program, Trent University, 1600 West Bank Drive, Peterborough, ON K9L 0G2, Canada; Department of Biology, Trent University, 1600 West Bank Drive, Peterborough, ON K9L 0G2, Canada; Department of Biology, Trent University, 1600 West Bank Drive, Peterborough, ON K9L 0G2, Canada

**Keywords:** Costs of reproduction, context-dependent trade-offs, dioecy, leaf nitrogen, life-history trade-offs, photosynthetic rates, sex-allocation theory, *Sagittaria latifolia*, sexual dimorphism, somatic costs of reproduction, source–sink dynamics, resource allocation

## Abstract

**Background and Aims:**

Trade-offs between current reproduction and future performance are fundamental constraints on evolution. In dioecious plants, females and males can differ in how much of a limiting resource they allocate to reproduction, creating trade-offs that could be sex specific and environmentally dependent. If so, physiological differences in resource use are expected to coincide with differential costs of reproduction for females versus males, but how these costs are expressed across environmental contexts remains poorly understood.

**Methods:**

We tested the hypothesis that the expression of sex-specific reproductive trade-offs is environmentally dependent in dioecious broadleaf arrowhead (*Sagittaria latifolia*). We conducted a 2-year common garden experiment manipulating nutrient availability and reproductive investment to quantify sex-specific physiological costs using photosynthetic measurements and indices of nitrogen content.

**Key Results:**

As expected, the expression of reproductive trade-offs depended on both sex and resource availability. At the lowest nutrient level, males experienced reproductive costs as reduced leaf nitrogen content and photosynthesis following flowering in year 1 and as reduced leaf nitrogen content and size in year 2. Across all nutrient conditions, females showed fewer detectable physiological costs.

**Conclusions:**

Together with results from previous studies of *S. latifolia*, our study shows that males and females incur reproductive costs in different physiological currencies, with males allocating more nitrogen than females. These costs become evident only under nitrogen limitation, highlighting how environmental context governs the expression of sex-specific life-history trade-offs.

## INTRODUCTION

The allocation of resources to current reproduction at the expense of future survival and growth is a fundamental trade-off in life-history evolution (Williams, 1966). These costs of reproduction are central to ecology and evolution, shaping phenomena as diverse as the evolution of delayed reproduction, the dynamics of herbivory and the persistence of clonal versus sexual life cycles (García and Ehrlén, 2002; Lin *et al.*, 2016; Kellett and Shefferson, 2018). Yet, how these costs are paid, and by which sex, might depend on the type of reproductive investment and the environmental context.

In dioecious flowering plants, female and male reproductive functions are distinct. Females invest in ovules, fruits and seeds, whereas males invest in pollen and structures for its dispersal (Lloyd, 1979). Because female structures are often larger, it has been widely assumed that females incur greater reproductive costs than males (Obeso, 2002). This assumption is frequently invoked to explain why males of many dioecious species exhibit faster growth or greater longevity (Delph, 1999; Barrett and Hough, 2013; Pannell, 2025). However, the view that sexual dimorphism is a direct consequence of greater female reproductive costs lacks broad empirical support (Dorken *et al.*, 2025) and overlooks the possibility that males and females pay for reproduction using different currencies (Dorken 2025).

Although fitness is the ultimate currency of reproductive costs (Williams, 1966; Bell, 1980), it is rarely measured directly, especially in long-lived plants (Calvo and Horvitz, 1990; Horvitz *et al.*, 2010; Miller *et al.*, 2012). Instead, research on reproductive costs often focuses on somatic costs: the immediate, negative impacts of reproduction on physiological performance, growth or survival, which serve as proxies for future fitness (Obeso, 2002; Dorken *et al.*, 2025). The expression of these somatic costs depends on the timing and type of resources invested. For example, nitrogen allocated to pollen production during flowering cannot simultaneously support photosynthetic enzymes, potentially limiting carbon gain and subsequent growth, particularly in nitrogen-poor environments (Anten *et al.*, 1995; Carmo-Silva *et al.*, 2015). If males and females rely on different resources allocated at different times, such as early-season nitrogen for pollen versus late-season carbon for fruit, realized costs of reproduction might be expressed differently between the sexes and be sensitive to resource availability (Seger and Eckhart, 1996; Eckhart and Seger, 1999). Despite its theoretical importance, empirical tests remain limited. An early experiment showed that male function can incur larger costs under nutrient limitation, possibly through nitrogen constraints (Eckhart and Chapin, 1997), but detailed mechanistic evidence has been scarce (Teitel *et al.*, 2016).

Here, we examined experimentally the hypothesis that resource availability mediates sex-specific somatic costs of reproduction. In a 2-year common garden experiment, we manipulated nutrient levels for female and male plants of the dioecious herb *Sagittaria latifolia* and prevented reproduction in a subset of individuals to establish a non-reproductive baseline. Previous work on *S. latifolia* showed that males allocated more nitrogen to reproduction than females and that photosynthesis is strongly nitrogen limited (Van Drunen and Dorken, 2012; Wright and Dorken, 2014). Based on this, we predicted that under nutrient limitation: (1) males would incur greater physiological costs than females, manifesting as reduced photosynthetic rates; and (2) these costs would translate into reduced growth in the following year. Conversely, we predicted that (3) under nutrient enrichment, these sex-specific costs would be ameliorated, reducing or eliminating sexual dimorphism in physiological performance and growth. We tested these predictions by quantifying somatic costs of reproduction using measurements of photosynthetic rates and leaf nitrogen content throughout the first growing season and assessed carry-over effects on plant growth in the following year. This approach allowed us to quantify dynamic trade-offs across a resource gradient, providing the first direct experimental evidence for the environmental control of sex-specific life-history trade-offs in plants.

## MATERIALS AND METHODS

### Study species and plant propagation

Broadleaf arrowhead (*Sagittaria latifolia* Willd.) is a clonal emergent aquatic plant native to a variety of wetland habitats in North America. This plant is unusual among angiosperms in that natural populations can be either dioecious or monoecious (Wooten, 1971; Dorken *et al.*, 2002). Plants produce a basal rosette of leaves, from which one or more clonal shoots (ramets) emerge. Flowering occurs from July to September. Male flowers produce pollen, whereas female flowers develop into fruits that disperse hundreds of achenes. The species reproduces sexually via seeds and asexually via the production of corms, which are underground perennating structures that sprout new ramets in the spring. At the end of the growing season, all vegetative biomass dies back, leaving seeds and corms as the only living plant tissue.

In February 2020, we collected open-pollinated seeds from 121 maternal plants across seven dioecious populations in southern Ontario (Supplementary Data Table S1). To minimize resampling of the same genet, seeds were collected from plants separated by ≥2 m. After a 3-week cold stratification period to break dormancy, seeds were germinated in a greenhouse at Trent University. Seedlings were grown in a 1:1 mixture of sand and peat-based potting soil, received weekly nutrient applications (20:20:20 N:P:K) to encourage establishment, and were transplanted into progressively larger pots over 12 weeks. In May 2020, we selected the largest 390 plants and moved them to an outdoor common garden in Lakehurst, ON, Canada. Because flower production in *S. latifolia* is positively correlated with initial plant size (Dorken and Barrett, 2004*a*), we selected large individuals to maximize the likelihood of flowering and thus enable sex determination. Although initial size influences flowering probability, its effect on final plant size is minimal in comparison to the strong influence of nutrient availability (Dorken and Barrett, 2004*a*). Each plant, in its own 20 cm (top diameter) pot, was placed into a 5 L bucket and watered to maintain a constant water level slightly below the soil surface. Plants were grown outdoors and frequently visited by pollinating insects; conditions that typically result in near-complete fruit set in *S. latifolia* (Dorken and Mitchard, 2008).

### Experimental design

We used a fully factorial, randomized design to manipulate nutrient availability and reproductive investment. Plants were assigned randomly to one of three nutrient treatments and one of two reproductive investment treatments (inflorescence intact or removed). Nutrient treatments consisted of a weekly 50 mL application of a low (0.03 %), medium (0.3 %) or high (3 %) solution of water-soluble 20:20:20 N:P:K fertilizer. These concentrations were chosen based on previous experiments to generate a range of plant sizes comparable to that observed in natural populations (Dorken and Mitchard, 2008; Van Drunen and Dorken, 2012). Nutrient applications began on 2 June and continued until 1 week after peak flowering in mid-August.

For the reproductive investment treatment, we either allowed inflorescences to develop normally (‘intact’) or removed them as soon as they emerged (‘removed’). As plants began to flower in mid-July, we recorded the sex of each individual. As expected under simple Mendelian inheritance (Dorken and Barrett, 2004*b*), the sex ratio was approximately equal, although males were more likely to flower under nutrient limitation, resulting in a final sample of 177 males and 107 females. A total of 53 plants, primarily from the low- and medium-nutrient treatments, failed to flower; their sex was unknown, and they were excluded from analyses.

### Measurements

#### Photosynthetic rates

We measured photosynthetic rates (net CO_2_ assimilation, in micromoles per metre squared per second) using a LI-6400XT portable gas exchange system (LI-COR Inc.) before (15 June), during (10 August) and after (10 September) peak flowering. First, to assess detailed photosynthetic physiology, we generated light-response curves for a random subset of ten intact plants (five female and five male) from each nutrient treatment at each time point. We fitted these curves to a non-rectangular hyperbola model (Marshall and Biscoe, 1980) to estimate the light-saturated photosynthetic rate (*A*_sat_), the light saturation point (*k*_sat_) and the curvature of the response (*θ*) (Supplementary Data Fig. S1; Evans and Santiago, 2014). Second, to assess broad physiological patterns, we measured light-saturated photosynthetic rates (*A*_sat_) on all experimental plants at each time point. All measurements were taken on the youngest, fully expanded leaf between 0800 and 1100 h in standardized chamber conditions (400 μmol CO_2_ mol^−1^, 30 °C, 65 % relative humidity).

#### Leaf nitrogen content

On the same dates as the *A*_sat_ measurements, we estimated leaf chlorophyll content using a SPAD 502 Plus meter (Minolta^®^). SPAD readings provide an index of chlorophyll content and are strongly correlated with leaf nitrogen in many species (Percival *et al.*, 2008; Mehrabi and Sepaskhah, 2022).

#### Post-reproductive growth

After senescence in October 2020, we harvested, counted and weighed all corms produced by each genet. To standardize comparisons and represent the most substantial reserve of resources for re-establishment, we planted the largest corm from each genet in March 2021. Owing to pandemic-related time constraints, initial corm mass was not recorded; instead, the largest corm was selected visually from pooled samples. Corms are physiologically independent structures (Supplementary Data Fig. S2) produced at the ends of long stolons (∼30 cm; Dorken and Barrett, 2004*a*), and each functions as a discrete store of resources for regrowth. This approach was intended to reflect the resource status of the ramet most likely to become re-established in the following year instead of total clonal investment, which was the focus of a previous study (Van Drunen and Dorken, 2012). Plants were grown for 75 days in a nutrient-free medium to estimate carry-over effects on size and leaf nitrogen content. At the end of the trial, we measured the mid-vein length of the largest leaf as an index of plant size (Sarkissian *et al.*, 2001) and recorded its SPAD value.

### Statistical analyses

All analyses were performed in R (v.4.5.0; R Core Team, 2025). We first analysed the light-response curve data using the photosynthesis package (Stinziano *et al.*, 2023) to estimate *A*_sat_, *k*_sat_ and *θ* for each combination of sex, nutrient treatment and flowering stage.

To test our main hypotheses, we used fixed-effects linear models (R function lm). Initially, we included source population as a random effect in mixed-effects models, but given that it did not improve model fit in any analysis, it was dropped. To analyse photosynthetic rates (*A*_sat_) and leaf nitrogen content (SPAD units) from 2020, we fitted a model with sex, nutrient treatment, reproductive treatment (intact/removed) and flowering stage (before/during/after) as fixed factors, including all higher-order interactions. To analyse subsequent growth (mid-vein length) and leaf nitrogen content (SPAD units) from 2021, we fitted a model with the 2020 treatments (sex, nutrient, reproductive) and their interactions as fixed factors.

For all models, we tested the significance of higher-order interactions using type III ANOVA using the Anova function in the car package (Fox and Weisberg, 2019). We interpreted significant interactions by conducting *post hoc* pairwise comparisons of least-squares means with the Tukey–Kramer method using the emmeans function in the emmeans package (Lenth, 2025).

## RESULTS

### Light-response curves

**Fig. 1.**
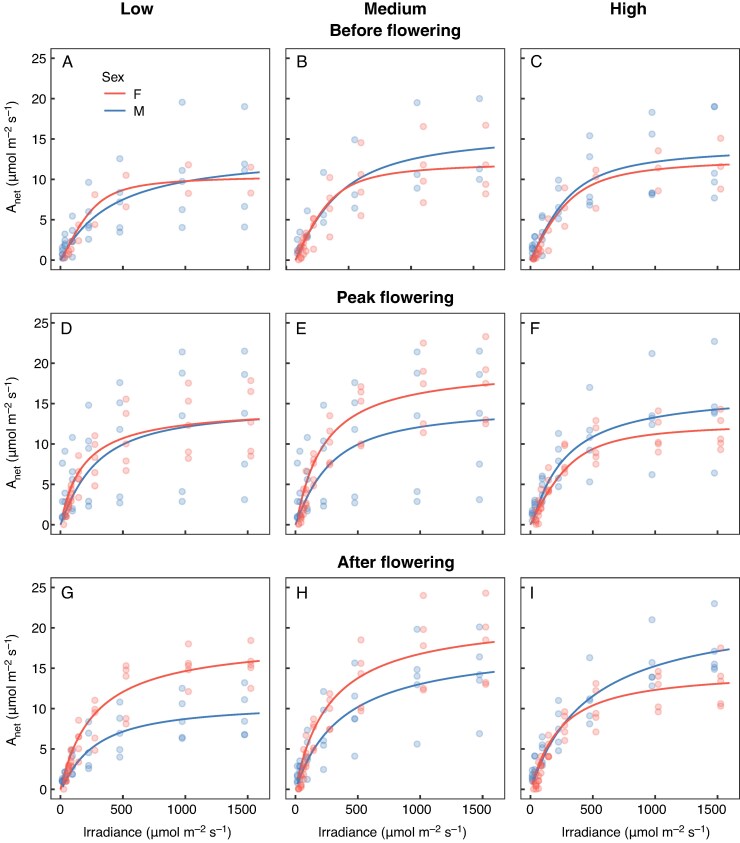
Light-response curves for female and male *Sagittaria latifolia* plants grown in a common garden before flowering (A–C), during peak flowering (D–F) and after flowering (G–I) and subjected to different levels of a nutrient-addition treatment. Arranged vertically, panels on the left (A, D, G) correspond to plants grown in low-nutrient conditions, panels in the middle (B, E, H) correspond to plants grown in medium-nutrient conditions, and panels on the right (C, F, I) correspond to plants grown in high-nutrient conditions. Points are individual estimates of net photosynthetic rates per tested level of irradiance, and curves are the fitted non-rectangular hyperbolas (Marshall and Biscoe, 1980; Stinziano *et al.*, 2023).

**Fig. 2.**
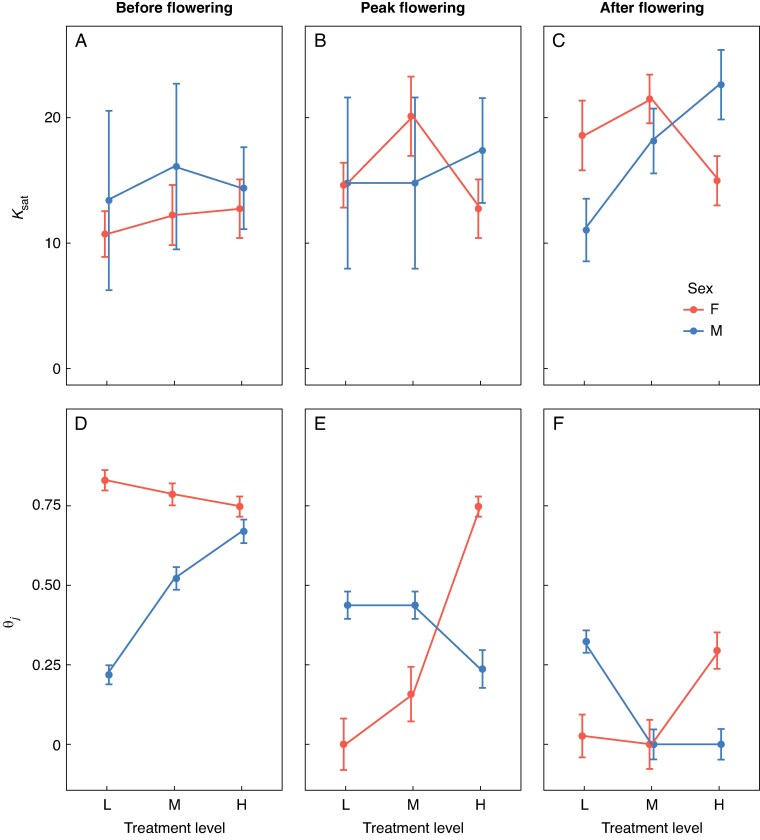
Inferred parameters for the light-response curves shown in Fig. 1 that indicate the light saturation point (*k*_sat_) and the degree of curvature (*θ*_j_) for female and male plants of *Sagittaria latifolia* grown in a common garden and subjected to a nutrient-addition treatment with three levels (low, medium and high). Panels are arranged vertically, with panels on the left corresponding to measures made before flowering (A, D), panels in the middle corresponding to measures made during peak flowering (B, E) and panels on the right corresponding to measures made after flowering (C, F).

The photosynthetic response to light differed between sexes, contingent on nutrient availability and flowering stage (Fig. 1). Before flowering, females in the low-nutrient treatment were more efficient at low light levels, exhibiting a light-response curvature (*θ*) more than three times greater than that of males (Fig. 2D), indicating that photosynthesis increased more rapidly with light in females in these conditions. This difference disappeared in high-nutrient conditions, where the sexes showed similar photosynthetic responses (Fig. 2D). After flowering, the pattern depended on nutrient status. In the low-nutrient treatment, the pattern was reversed for most aspects of photosynthetic performance. Males exhibited substantially lower light-saturated photosynthetic rates (*A*_sat_; Fig. 1G), indicating reduced maximum photosynthetic capacity. They also had a lower light saturation point (*k*_sat_; Fig. 2C), meaning that photosynthesis reached its maximum at lower light levels in males compared with females, and greater curvature (*θ*; Fig. 2F), meaning a steeper initial increase in photosynthesis with light compared with females. In contrast, in high-nutrient conditions, post-flowering females had lower photosynthetic performance than males. Females showed: (1) lower photosynthetic rates at high light levels (*A*_sat_; Fig. 1I), indicating reduced maximum photosynthetic capacity; (2) a lower light saturation point (*k*_sat_; Fig. 2C), meaning that photosynthesis reached its maximum at lower light levels; and (3) greater curvature (*θ*; Fig. 2F), meaning a steeper initial increase in photosynthesis with light compared with males.

### Light-saturated photosynthesis

**Fig. 3.**
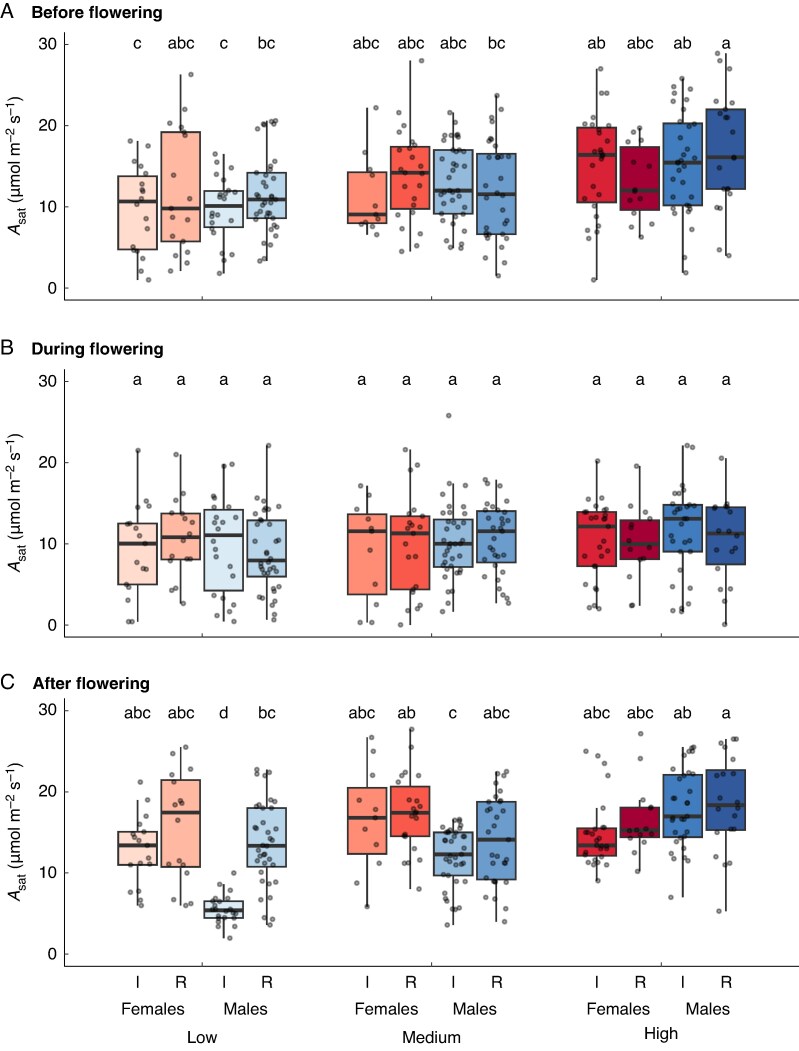
Variation in rates of light-saturated photosynthesis (*A*_sat_) before flowering (A), during peak flowering (B) and after flowering (C) for female and male plants of *Sagittaria latifolia* grown in a common garden and subjected to a nutrient-addition treatment with three levels (low, medium and high) and an inflorescence-removal treatment, for which all flowers were either removed (R) or left intact (I). The boxes indicate the median values of *A*_sat_ for each plant sex and treatment combination using a solid black line in the interior of each box. The top and bottom of each box indicate the upper and lower quartiles, and whiskers extend to 1.5× the upper and lower interquartile range. Letters above each box indicate the results of a Tukey–Kramer *post hoc* analysis; pairs of boxes that do not share a letter are significantly different.

Analysis of *A*_sat_ across all plants supported the findings from the light-response curves, revealing a significant three-way interaction between flowering stage, nutrient level and sex (Supplementary Data Table S2). Before and during flowering, *A*_sat_ was similar for both sexes across all nutrient treatments (Fig. 3A, B). The key difference emerged after flowering: in the low-nutrient treatment, intact (reproducing) males had significantly lower *A*_sat_ than both intact females and non-reproducing (inflorescence-removed) males (Fig. 3C). This cost of reproduction for males was not apparent in the high-nutrient treatment, where post-flowering *A*_sat_ was similar for both sexes regardless of reproductive status (Fig. 3C).

### Leaf nitrogen content

**Fig. 4.**
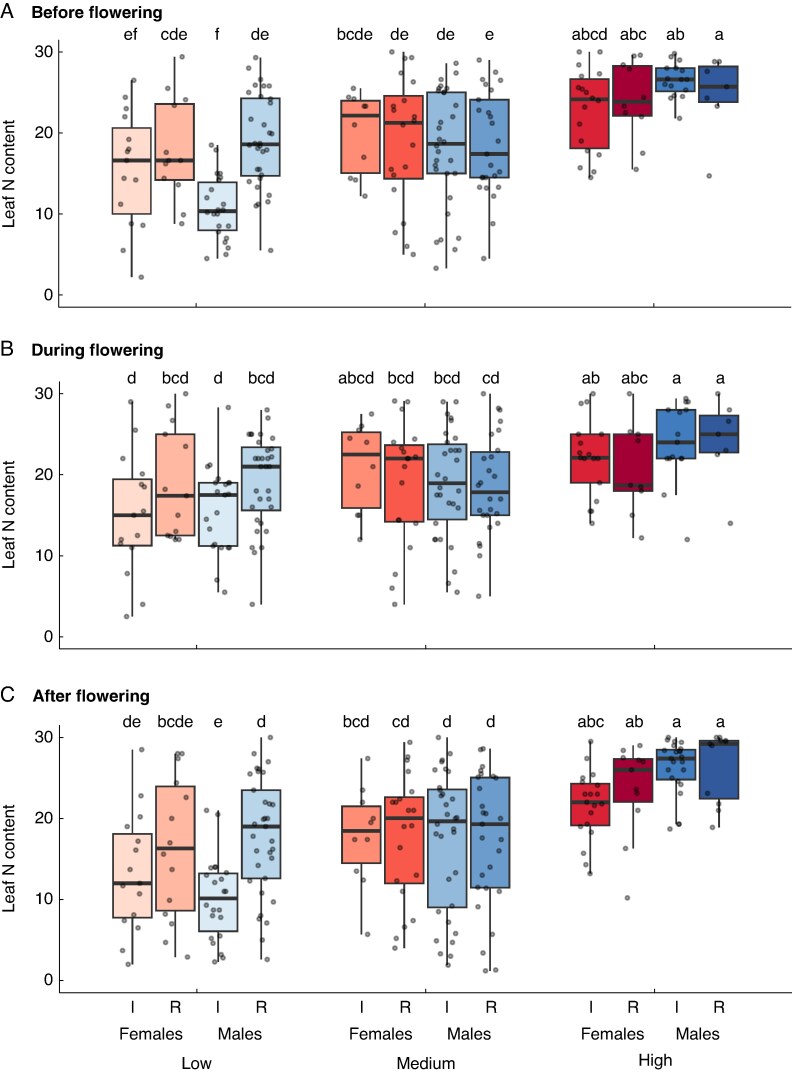
Variation in leaf chlorophyll content (in SPAD units, used as an index of leaf nitrogen content). Measurements were made before flowering (A), during peak flowering (B) and after flowering (C) for female and male plants of *Sagittaria latifolia* grown in a common garden and subjected to a nutrient-addition treatment with three levels (low, medium and high) and an inflorescence-removal treatment, for which all flowers were either removed (R) or left intact (I). The solid black line in the interior of each box indicates the median value of leaf nitrogen content for each plant sex and treatment combination. The top and bottom of each box indicate the upper and lower quartiles, and whiskers extend to 1.5× the upper and lower interquartile range. Letters above each box indicate the results of a Tukey–Kramer *post hoc* analysis; pairs of boxes that do not share a letter are significantly different.

Patterns of leaf nitrogen content (SPAD values) mirrored those of photosynthesis (Supplementary Data Table S3). Although estimated leaf nitrogen content was broadly similar between sexes before and during flowering, a cost of reproduction was evident for males after flowering. In the low-nutrient treatment, intact males had significantly lower SPAD values than their non-reproducing counterparts (Fig. 4C). This difference was absent in the medium- and high-nutrient treatments, where post-flowering SPAD values were similar for all groups (Fig. 4C).

### Post-reproductive growth

**Fig. 5.**
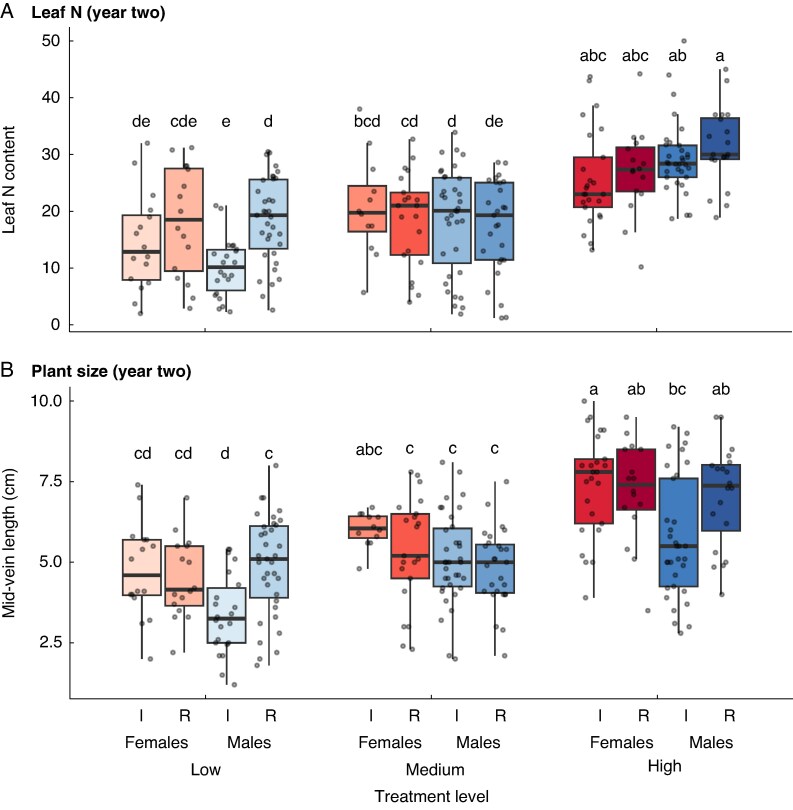
Variation in leaf chlorophyll content (in SPAD units, used as an index of leaf nitrogen content; A); and mid-vein length (used as an index of plant size; B) among female and male plants of *Sagittaria latifolia* that had been grown in a common garden experiment in the previous year. Plants in the common garden were subjected to a nutrient-addition treatment with three levels (low, medium and high) and an inflorescence-removal treatment, for which all flowers were either removed (R) or left intact (I). The solid black line in the interior of each box indicates the median values of leaf nitrogen content and plant size for each plant sex and treatment combination. The top and bottom of each box indicate the upper and lower quartiles, and whiskers extend to 1.5× the upper and lower interquartile range. Letters above each box indicate the results of a Tukey–Kramer *post hoc* analysis; pairs of boxes that do not share a letter are significantly different.

The physiological costs observed in 2020 carried over to affect plant growth and nutrient status in 2021. After 75 days of growth without supplemental nutrients, plants from the low-nutrient treatment showed a significant interaction between sex and the 2020 reproductive treatment (Supplementary Data Table S4). Males that had reproduced in 2020 had significantly lower estimated leaf nitrogen content (SPAD values) than males whose inflorescences had been removed (Fig. 5A). This effect was not observed in females or in plants from the higher-nutrient treatments. Likewise, plant size in 2021 was affected by a significant interaction between sex and the 2020 reproductive treatment, with males that reproduced in low-nutrient conditions tending to be smaller than their non-reproducing counterparts (Fig. 5B).

## DISCUSSION

### Sex- and environment-specific costs of reproduction

Our results provide clear experimental evidence that the somatic costs of reproduction in *S. latifolia* are both sex specific and dependent on resource availability. These findings align with theoretical predictions that sex-specific costs depend on resource allocation and environmental context (Seger and Eckhart, 1996) and extend early experimental evidence that male function can incur greater costs under nutrient limitation (Eckhart and Chapin, 1997) by identifying nitrogen-based physiological mechanisms underlying these patterns. Under nutrient limitation, reproducing males incurred significant physiological and growth trade-offs that were not observed in females. Conversely, under nutrient enrichment, these costs were undetectable in either sex. These findings complement previous work showing that females experience biomass-based trade-offs between reproduction and end-of-season size (Van Drunen and Dorken, 2012). Our study focused on nitrogen-sensitive physiological traits, and our results should not be interpreted as evidence that females incur no costs of reproduction; rather, females might pay costs in other currencies, such as clonal growth or corm biomass, as shown in previous studies (Van Drunen and Dorken, 2012). Importantly, our estimates of post-reproductive performance reflect trade-offs expressed within individual ramets, rather than across all ramets within a genet, and thus capture a different dimension of reproductive cost. We now show that nitrogen-driven physiological costs (declines in photosynthesis and leaf nitrogen) emerge in males only under nutrient limitation, highlighting that the sex incurring the greater cost can shift with environmental conditions.

Recognizing that reproductive costs are paid in different, environmentally sensitive currencies helps to resolve conflicting findings from a prior study. A field study of *S. latifolia* in a nutrient-rich agricultural ditch found no difference in photosynthetic rates between the sexes (Wright and Dorken, 2014). Our results explain this by demonstrating that high nutrient availability can mask the underlying physiological costs of reproduction, particularly for males. This difference between allocation and the expression of reproductive costs underscores an important point: reproductive costs are not equivalent to reproductive allocations (Dorken *et al.*, 2025). Specifically, reproductive allocations (e.g. estimates of reproductive effort) are not estimates of reproductive costs; an allocation is an investment that yields a current fitness benefit, whereas a cost is the resulting reduction in future fitness (Bell, 1980). As our study shows, this relationship is not fixed; plants in our high-nutrient treatment made substantial reproductive allocations but incurred no detectable somatic cost, demonstrating that the function relating allocation to cost is environmentally dependent.

### Trade-offs between reproduction and photosynthesis

Reproduction can alter leaf-level physiology through changes in source–sink dynamics. High carbon demand from developing fruits can upregulate photosynthesis in nearby source leaves (Gifford and Evans, 1981). Conversely, experimental fruit removal can decrease photosynthetic rates, as has been shown in soybeans, for example (Setter *et al.*, 1980). We found no evidence for photosynthetic downregulation among non-reproducing *S. latifolia* females, probably because all females, regardless of whether they produced fruits, invest heavily in an alternative sink: overwintering corms (Van Drunen and Dorken, 2012). As a result, the total sink strength might not have differed substantially between reproducing and non-reproducing females in our experiment.

More striking was the significant photosynthetic downregulation in reproducing males. The substantial post-reproductive drop in photosynthetic capacity among males found here contrasts with studies of other dioecious species. For example, for *Silene latifolia* males often have higher photosynthetic rates than females, a pattern attributed to differences in leaf morphology rather than nitrogen content (Delph *et al.*, 2005). In *Sagittaria latifolia*, the mechanism appears to be different. Photosynthesis is strongly tied to leaf nitrogen (Wright and Dorken, 2014), and our results are consistent with significant depletion of leaf nitrogen in nutrient-poor conditions in males, but not females. This nitrogen-based physiological cost appears to represent a key trade-off associated with male function in this species and could be detected only by comparing reproducing and non-reproducing plants across a resource-availability gradient. This might also explain why greater costs of reproduction for males compared with females are detected less often than the reverse (Dorken *et al.*, 2025); if experimental studies are conducted in benign conditions, trade-offs involving nutrients can be masked.

### Implications of sex-specific costs of reproduction

Our findings identify resource limitation as a key factor shaping sex-specific reproductive costs, with potential consequences for population-level traits, such as sex ratio, especially when interacting with broader environmental constraints. For example, populations of *Sagittaria latifolia* at the northern range limit are typically male biased, a pattern thought to be driven by greater resource constraints on females (Yakimowski and Barrett, 2014). For this plant, females experience stronger carbon-based trade-offs, allocating less biomass to clonal propagules than males (Van Drunen and Dorken, 2012). If shorter growing seasons at higher latitudes restrict carbon assimilation, females might incur greater reproductive costs, leading to male-biased sex ratios. More broadly, sex-specific reproductive costs might underlie geographical variation in sex ratios across dioecious plants, which are often correlated with latitude, elevation and resource availability (Field *et al.*, 2013). Indeed, because sex-specific reproductive costs influence key demographic parameters (growth, survival and reproduction) their effects should extend beyond sex ratios to impact population viability and geographical distributions of dioecious plants.

### The nature of reproductive costs in dioecious plants

Together with previous research on *S. latifolia* (Van Drunen and Dorken, 2012), our findings show that males and females pay for reproduction with different physiological currencies, a pattern that has broad implications for models of sex allocation and the evolution of dioecy. Our measure of year 2 size reflects growth from the largest corm rather than total corm biomass, providing a standardized proxy for genet resource status but limiting detection of trade-offs at the level of clonal investment (Van Drunen and Dorken, 2012). Life-history models often assume that female and male functions draw from a common resource pool (Charnov *et al.*, 1976; Charlesworth and Morgan, 1991; Crowley, 2008). Our results challenge this assumption. The fact that reproduction depleted leaf nitrogen in males but not females, for example, is inconsistent with a single, shared resource pool. Models that incorporate sex-specific resource currencies predict contrasting equilibrium allocations to female and male function in hermaphrodites and shifts in sex ratios in sexually dimorphic plants (Venable and Lloyd, 2004; Dorken and Van Drunen, 2018). Our findings support evidence from studies of diverse dioecious plants showing that males and females do not draw from equivalent, interchangeable resource pools (Eckhart and Chapin, 1997; Nicotra, 1999; Nicotra and Leigh, 2003; Delph *et al.*, 2005; Harris and Pannell, 2008; Teitel *et al.*, 2016). Accordingly, models of sex allocation would more accurately reflect ecological constraints by treating sex-specific resource currencies as the default assumption rather than assuming a shared resource pool.

Recently, it has been argued that because average female and male fitness must be equal in a stable population, their fitness costs of reproduction must also be equal (Midgley, 2022). Our results provide direct, mechanistic evidence against this view on two fronts. First, equal average fitness does not require identical life histories (Pannell, 2025); the constraint of equal fitness does not prevent the evolution of divergent schedules of growth, survival and reproduction between the sexes (Dorken, 2025). Second, as noted above, resource allocation is not equivalent to reproductive cost. Because males and females pay for reproduction with different currencies, the expression of their respective costs can differ. Our study adds to this conclusion by providing evidence that sex-specific costs not only exist but are shaped by the ecological context in which they occur.

## Supplementary Material

mcaf296_Supplementary_Data

## Data Availability

Data and R codes from this study are available at figshare: https://doi.org/10.6084/m9.figshare.29403653.v1.

## SUPPLEMENTARY DATA

Table S1: locality information for the seven source populations used to obtain material for this experiment. Table S2: ANOVA for the effects of nutrient treatment, reproductive investment (inflorescence removal), flowering stage, sex and their interactions on light-saturated photosynthetic rates (*A*_sat_). Table S3: ANOVA for the effects of nutrient treatment, reproductive investment (inflorescence removal), flowering stage, sex and their interactions on leaf nitrogen content. Table S4: ANOVA for the effects of nutrient treatment, reproductive investment (inflorescence removal), sex and their interactions on the size of plants in the year after reproduction. Figure S1: diagram describing the three photosynthetic parameters estimated in this study (*k*_sat_, *θ*_j_ and *A*_sat_). Figure S2: photographs of female and male plants from year 1 of this study. The corms shown in the lower-right panel were collected from a single plant grown in the 2020 common garden.

## AI ASSISTANCE

AI-assisted technologies were used to refine the text of a previous draft of the manuscript. AI-assisted technologies were not used to generate original text or for any other component of the research described in this study.

## References

[mcaf296-B1] Anten NPR, Schieving F, Werger MJA. 1995. Patterns of light and nitrogen distribution in relation to whole canopy carbon gain in C_3_ and C_4_ mono- and dicotyledonous species. Oecologia 101: 504–513. doi:10.1007/BF00329431 28306967

[mcaf296-B2] Barrett SCH, Hough J. 2013. Sexual dimorphism in flowering plants. Journal of Experimental Botany 64: 67–82. doi:10.1093/jxb/ers308 23183260

[mcaf296-B3] Bell G . 1980. The costs of reproduction and their consequences. The American Naturalist 116: 45–76. doi:10.1086/283611

[mcaf296-B4] Calvo RN, Horvitz CC. 1990. Pollinator limitation, cost of reproduction, and fitness in plants: a transition matrix approach. The American Naturalist 136: 499–516. doi:10.1086/285110

[mcaf296-B5] Carmo-Silva E, Scales JC, Madgwick PJ, Parry MAJ. 2015. Optimizing Rubisco and its regulation for greater resource use efficiency. Plant, Cell & Environment 38: 1817–1832. doi:10.1111/pce.12425 25123951

[mcaf296-B6] Charlesworth D, Morgan MT. 1991. Allocation of resources to sex functions in flowering plants. Philosophical Transactions of the Royal Society B: Biological Sciences 332: 91–102. doi:10.1098/rstb.1991.0036

[mcaf296-B7] Charnov EL, Bull JJ, Smith JM. 1976. Why be an hermaphrodite? Nature 263: 125–126. doi:10.1038/263125a0

[mcaf296-B8] Crowley PH . 2008. Sex allocation in simultaneous hermaphrodites: trade-offs between sex-specific costs and lifespan. Theoretical Ecology 1: 199–208. doi:10.1007/s12080-008-0020-6

[mcaf296-B9] Delph LF . 1999. Sexual dimorphism in life history. In: Geber MA, Dawson TE, Delph LF. eds. Gender and sexual dimorphism in flowering plants. Berlin: Springer, 149–173.

[mcaf296-B10] Delph LF, Gehring JL, Arntz AM, Levri M, Frey FM. 2005. Genetic correlations with floral display lead to sexual dimorphism in the cost of reproduction. The American Naturalist 166: S31–S41. doi:10.1086/444597 16224710

[mcaf296-B11] Dorken ME . 2025. Do costs of reproduction differ between the sexes of dioecious plants? Annals of Botany mcaf178. doi:10.1093/aob/mcaf178 PMC12823230 40795309

[mcaf296-B12] Dorken ME, Barrett SCH. 2004a. Phenotypic plasticity of vegetative and reproductive traits in monoecious and dioecious populations of *Sagittaria latifolia* (Alismataceae): a clonal aquatic plant. The Journal of Ecology 92: 32–44. doi:10.1111/j.1365-2745.2004.00857.x

[mcaf296-B13] Dorken ME, Barrett SCH. 2004b. Sex determination and the evolution of dioecy from monoecy in *Sagittaria latifolia* (Alismataceae). Proceedings of the Royal Society B: Biological Sciences 271: 213–219. doi:10.1098/rspb.2003.2580 PMC1691578 15058400

[mcaf296-B14] Dorken ME, Friedman J, Barrett SCH. 2002. The evolution and maintenance of monoecy and dioecy in *Sagittaria latifolia* (Alismataceae). Evolution; International Journal of Organic Evolution 56: 31–41. doi:10.1111/j.0014-3820.2002.tb00847.x 11915852

[mcaf296-B15] Dorken ME, Mitchard ETA. 2008. Phenotypic plasticity of hermaphrodite sex allocation promotes the evolution of separate sexes: an experimental test of the sex-differential plasticity hypothesis using *Sagittaria latifolia* (Alismataceae). Evolution; International Journal of Organic Evolution 62: 971–978. doi:10.1111/j.1558-5646.2008.00336.x 18248634

[mcaf296-B16] Dorken ME, Van Drunen WE. 2018. Life-history trade-offs promote the evolution of dioecy. Journal of Evolutionary Biology 31: 1405–1412. doi:10.1111/jeb.13335 29908091

[mcaf296-B17] Dorken ME, van Kleunen M, Stift M. 2025. Costs of reproduction in flowering plants. New Phytologist 247: 55–70. doi:10.1111/nph.70166 40342244 PMC12138173

[mcaf296-B18] Eckhart VM, Chapin FS III. 1997. Nutrient sensitivity of the cost of male function in gynodioecious *Phacelia linearis* (Hydrophyllaceae). American Journal of Botany 84: 1092–1098. doi:10.2307/2446152 21708664

[mcaf296-B19] Eckhart VM, Seger J. 1999. Phenological and developmental costs of male sex function. In: Vuorisalo T, Mutikainen P. eds. Life history evolution in plants. Dordrecht: Kluwer Academic, 195–213.

[mcaf296-B20] Evans JR, Santiago LS. 2014. PrometheusWiki gold leaf protocol: gas exchange using LI-COR 6400. Functional Plant Biology: FPB 41: 223–226. doi:10.1071/FP10900 32480982

[mcaf296-B21] Field DL, Pickup M, Barrett SCH. 2013. Ecological context and metapopulation dynamics affect sex-ratio variation among dioecious plant populations. Annals of Botany 111: 917–923. doi:10.1093/aob/mct040 23444124 PMC3631328

[mcaf296-B22] Fox J, Weisberg S. 2019. An R companion to applied regression. Thousand Oaks, CA: Sage.

[mcaf296-B23] García MB, Ehrlén J. 2002. Reproductive effort and herbivory timing in a perennial herb: fitness components at the individual and population levels. American Journal of Botany 89: 1295–1302. doi:10.3732/ajb.89.8.1295 21665732

[mcaf296-B24] Gifford RM, Evans LT. 1981. Photosynthesis, carbon partitioning, and yield. Annual Review of Plant Physiology 32: 485–509. doi:10.1146/annurev.pp.32.060181.002413

[mcaf296-B25] Harris MS, Pannell JR. 2008. Roots, shoots and reproduction: sexual dimorphism in size and costs of reproductive allocation in an annual herb. Proceedings of the Royal Society B: Biological Sciences 275: 2595–2602. doi:10.1098/rspb.2008.0585 PMC2605799 18682371

[mcaf296-B26] Horvitz C, Ehrlen J, Matlaga D. 2010. Context-dependent pollinator limitation in stochastic environments: can increased seed set overpower the cost of reproduction in an understorey herb? The Journal of Ecology 98: 268–278. doi:10.1111/j.1365-2745.2009.01628.x

[mcaf296-B27] Kellett KM, Shefferson RP. 2018. Temporal variation in reproductive costs and payoffs shapes the flowering strategy of a neotropical milkweed, *Asclepias curassavica*. Population Ecology 60: 77–87. doi:10.1007/s10144-018-0618-5

[mcaf296-B28] Lenth RV . 2025. *Emmeans: estimated marginal means, aka least-squares means*. R package version 1.10.0. doi:10.32614/CRAN.package.emmeans.

[mcaf296-B29] Lin C-H, Miriti MN, Goodell K. 2016. Demographic consequences of greater clonal than sexual reproduction in *Dicentra canadensis*. Ecology and Evolution 6: 3871–3883. doi:10.1002/ece3.2163 27247759 PMC4867665

[mcaf296-B30] Lloyd DG . 1979. Parental strategies of angiosperms. New Zealand Journal of Botany 17: 595–606. doi:10.1080/0028825X.1979.10432573

[mcaf296-B31] Marshall B, Biscoe PV. 1980. A model for C_3_ leaves describing the dependence of net photosynthesis on irradiance: II. Application to the analysis of flag leaf photosynthesis. Journal of Experimental Botany 31: 41–48. doi:10.1093/jxb/31.1.41

[mcaf296-B32] Mehrabi F, Sepaskhah AR. 2022. Leaf nitrogen, based on SPAD chlorophyll reading can determine agronomic parameters of winter wheat. International Journal of Plant Production 16: 77–91. doi:10.1007/s42106-021-00172-2

[mcaf296-B33] Midgley JJ . 2022. The costs of reproduction in plants cannot differ between the sexes. Journal of Plant Ecology 15: 1308–1311. doi:10.1093/jpe/rtac103

[mcaf296-B34] Miller TEX, Williams JL, Jongejans E, Brys R, Jacquemyn H. 2012. Evolutionary demography of iteroparous plants: incorporating non-lethal costs of reproduction into integral projection models. Proceedings of the Royal Society B: Biological Sciences 279: 2831–2840. doi:10.1098/rspb.2012.0326 PMC3367791 22418255

[mcaf296-B35] Nicotra AB . 1999. Reproductive allocation and the long-term costs of reproduction in *Siparuna grandiflora*, a dioecious neotropical shrub. Journal of Ecology 87: 138–149. doi:10.1046/j.1365-2745.1999.00337.x

[mcaf296-B36] Nicotra AB, Leigh A. 2003. Sexual dimorphism in reproductive allocation and water-use efficiency in *Maireana pyramidata* (Chenopodiaceae), a dioecious, semi-arid shrub. Australian Journal of Botany 51: 509–514. doi:10.1071/BT03043

[mcaf296-B37] Obeso JR . 2002. The costs of reproduction in plants. New Phytologist 155: 321–348. doi:10.1046/j.1469-8137.2002.00477.x 33873312

[mcaf296-B38] Pannell JR . 2025. The costs of reproduction can and do differ between the sexes. Annals of Botany mcaf073. doi:10.1093/aob/mcaf073 PMC12718004 40255013

[mcaf296-B39] Percival G, Keary I, Noviss K. 2008. The potential of a chlorophyll content SPAD meter to quantify nutrient stress in foliar tissue of sycamore (*Acer pseudoplatanus*), English oak (*Quercus robur*), and European beech (*Fagus sylvatica*). Arboriculture & Urban Forestry 34: 89–100. doi:10.48044/jauf.2008.012

[mcaf296-B40] R Core Team . 2025. R: a language and environment for statistical computing. Vienna, Austria: R Foundation for Statistical Computing.

[mcaf296-B41] Sarkissian TS, Barrett SCH, Harder LD. 2001. Gender variation in *Sagittaria latifolia* (Alismataceae): is size all that matters? Ecology 82: 360–373. doi:10.1890/0012-9658(2001)082[0360:GVISLA]2.0.CO;2

[mcaf296-B42] Seger J, Eckhart VM. 1996. Evolution of sexual systems and sex allocation in plants when growth and reproduction overlap. Proceedings of the Royal Society B: Biological Sciences 263: 833–841. doi:10.1098/rspb.1996.0123

[mcaf296-B43] Setter TL, Brun WA, Brenner ML. 1980. Stomatal closure and photosynthetic inhibition in soybean leaves induced by petiole girdling and pod removal. Plant Physiology 65: 884–887. doi:10.1104/pp.65.5.884 16661301 PMC440443

[mcaf296-B44] Stinziano JR, Roback C, Gamble D, Murphy BK, Hudson PJ, Muir CD. 2023. *Photosynthesis: tools for plant ecophysiology & modeling*. R package version 2.1.0. 10.32614/CRAN.package.photosynthesis.

[mcaf296-B45] Teitel Z, Pickup M, Field DL, Barrett SCH. 2016. The dynamics of resource allocation and costs of reproduction in a sexually dimorphic, wind-pollinated dioecious plant. Plant Biology 18: 98–103. doi:10.1111/plb.12336 25865555

[mcaf296-B46] Van Drunen WE, Dorken ME. 2012. Trade-offs between clonal and sexual reproduction in *Sagittaria latifolia* (Alismataceae) scale up to affect the fitness of entire clones. New Phytologist 196: 606–616. doi:10.1111/j.1469-8137.2012.04260.x 22897332

[mcaf296-B47] Venable DL, Lloyd DG. 2004. Allocation under multiple resource constraints. Evolutionary Ecology Research 6: 1109–1121.

[mcaf296-B48] Williams GC . 1966. Natural selection, the costs of reproduction, and a refinement of Lack’s principle. The American Naturalist 100: 687–690. doi:10.1086/282461

[mcaf296-B49] Wooten JW . 1971. The monoecious and dioecious conditions in *Sagittaria latifolia* L. (Alismataceae). Evolution; International Journal of Organic Evolution 25: 549–553. doi:10.1111/j.1558-5646.1971.tb01915.x 28565022

[mcaf296-B50] Wright VL, Dorken ME. 2014. Sexual dimorphism in leaf nitrogen content but not photosynthetic rates in *Sagittaria latifolia* (Alismataceae). Botany 92: 109–112. doi:10.1139/cjb-2013-0246

[mcaf296-B51] Yakimowski S, Barrett S. 2014. Variation and evolution of sex ratios at the northern range limit of a sexually polymorphic plant. Journal of Evolutionary Biology 27: 1454–1466. doi:10.1111/jeb.12322 24506681

