# Supplementary material for: Environmental context shapes sex-specific costs of reproduction in a dioecious plant
Source: Ann Bot. 2025 Nov 14;137(4):1036–46. doi: 10.1093/aob/mcaf296 (PMC13095889; doi:10.1093/aob/mcaf296)
Supplement: mcaf296_Supplementary_Data [file mcaf296_supplementary_data.zip › FigS1_GeneralizedLightResponseCurve.pdf]

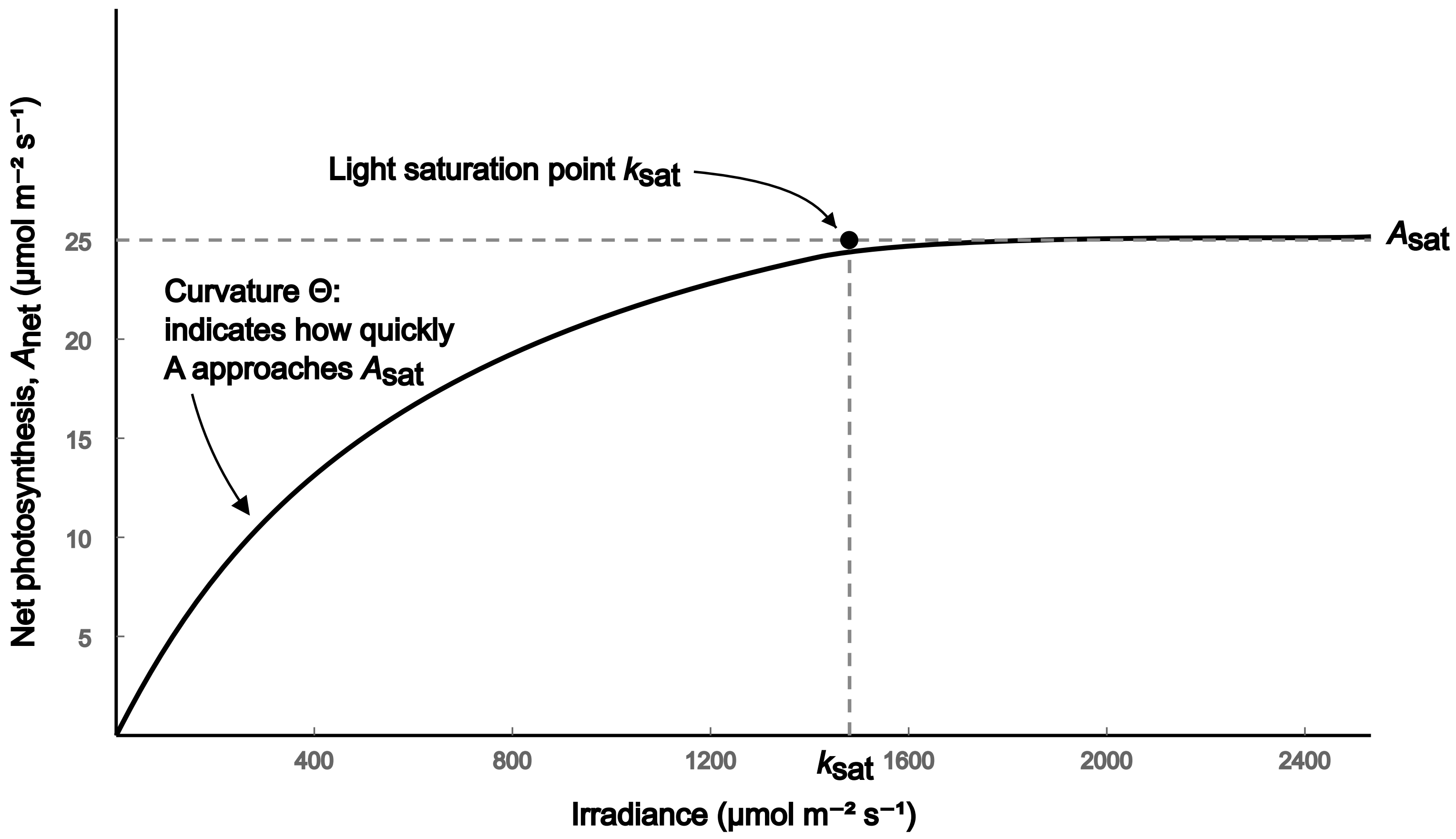

Figure S1. Generalized diagram indicating the three photosynthetic parameters estimated in this study. The curvature parameter (Θ) indicates how quickly the CO_2 assimilation rate approaches (*A*) A_{sat}. The light saturation point (k_{sat}) indicates the light intensity at which the net photosynthetic rate reaches its maximum and plateaus. Finally, A_{sat} is the net CO_2 assimilation rate under light-saturating conditions.
