# Supplementary material for: Environmental context shapes sex-specific costs of reproduction in a dioecious plant
Source: Ann Bot. 2025 Nov 14;137(4):1036–46. doi: 10.1093/aob/mcaf296 (PMC13095889; doi:10.1093/aob/mcaf296)
Supplement: mcaf296_Supplementary_Data [file mcaf296_supplementary_data.zip › TableS1.docx]

**Table S1**. Locality information for the seven source populations of *Sagittaria latifolia*, including the number of maternal seed families used for generating the maternal seed used in the experiment.

| **Site Location** | **Site Code** | **Latitude** | **Longitude** | ***n*** | **Habitat** |
| --- | --- | --- | --- | --- | --- |
| **Actinolite** | ATL | N44.54853 | W77.32333 | 32 | shallow pond |
| **Deerbrook** | DER | N42.29376 | W82.62192 | 64 | agricultural ditch |
| **Ecole St. Paul** | ESP | N42.30619 | W82.54784 | 98 | roadside ditch |
| **Long Point** | LTP | N42.58022 | W80.44023 | 68 | roadside ditch |
| **Point Pelee** | PPL | N42.00621 | W82.49547 | 47 | agricultural ditch |
| **Stoney Point** | STP | N42.30486 | W82.53218 | 25 | roadside ditch |
| **Stromness** | STB | N42.88404 | W79.55177 | 51 | shallow pond |
