# Supplementary material for: Environmental context shapes sex-specific costs of reproduction in a dioecious plant
Source: Ann Bot. 2025 Nov 14;137(4):1036–46. doi: 10.1093/aob/mcaf296 (PMC13095889; doi:10.1093/aob/mcaf296)
Supplement: mcaf296_Supplementary_Data [file mcaf296_supplementary_data.zip › TableS2.docx]

Table S2. Analysis of Variance (ANOVA) for the effects of nutrient treatment, reproductive investment (inflorescence removal), flowering stage, sex, and their interactions on light-saturated photosynthetic rates (*A*_sat_) in dioecious *Sagittaria latifolia* grown in a common garden. Values shown are the parameter estimates, their confidence intervals, and *p*-values. Fixed effects in the model were: Nutrient treatment (Nutr) with three levels: low, medium, highl Reproductive investment (Repr) with two levels: intact, and removed (Rem); flowering stage with three levels: before, during, after; and sex with two levels: female and male (M).

|  | ***A*_sat_** | | |
| --- | --- | --- | --- |
| *Predictors* | *Estimates* | *CI* | *p* |
| (Intercept) | 9.73 | 7.24 – 12.23 | **<0.001** |
| Nutr [Medium] | 1.64 | -2.41 – 5.70 | 0.427 |
| Nutr [High] | 5.44 | 2.22 – 8.67 | **0.001** |
| Repr [Rem] | 2.26 | -1.32 – 5.84 | 0.216 |
| Stage [During] | -0.39 | -3.97 – 3.19 | 0.831 |
| Stage [After] | 3.09 | -0.49 – 6.68 | 0.090 |
| Sex [M] | 0.00 | -3.36 – 3.37 | 0.998 |
| Nutr [Medium] × Repr [Rem] | 0.32 | -4.97 – 5.60 | 0.906 |
| Nutr [High] × Repr [Rem] | -3.26 | -8.21 – 1.68 | 0.196 |
| Nutr [Medium] × Stage [During] | -1.78 | -7.55 – 3.99 | 0.545 |
| Nutr [High] × Stage [During] | -4.37 | -8.97 – 0.23 | 0.063 |
| Nutr [Medium] × Stage [After] | 2.08 | -3.68 – 7.85 | 0.479 |
| Nutr [High] × Stage [After] | -3.43 | -8.03 – 1.17 | 0.144 |
| Repr [Rem] × Stage [During] | -0.88 | -6.02 – 4.26 | 0.737 |
| Repr [Rem] × Stage [After] | 0.62 | -4.53 – 5.76 | 0.814 |
| Nutr [Medium] × Sex [M] | 1.70 | -3.26 – 6.66 | 0.501 |
| Nutr [High] × Sex [M] | 0.20 | -4.16 – 4.56 | 0.929 |
| Repr [Rem] × Sex [M] | -0.37 | -4.94 – 4.21 | 0.876 |
| Stage [During] × Sex [M] | 0.53 | -4.27 – 5.33 | 0.827 |
| Stage [After] × Sex [M] | -7.28 | -12.08 – -2.48 | **0.003** |
| (Nutr [Medium] × Repr [Rem]) × Stage [During] | -1.46 | -9.00 – 6.08 | 0.703 |
| (Nutr [High] × Repr [Rem]) × Stage [During] | 1.59 | -5.50 – 8.68 | 0.659 |
| (Nutr [Medium] × Repr [Rem]) × Stage [After] | -2.37 | -9.91 – 5.17 | 0.538 |
| (Nutr [High] × Repr [Rem]) × Stage [After] | 2.16 | -4.93 – 9.25 | 0.550 |
| (Nutr [Medium] × Repr [Rem]) × Sex [M] | -3.24 | -9.76 – 3.29 | 0.331 |
| (Nutr [High] × Repr [Rem]) × Sex [M] | 3.04 | -3.40 – 9.48 | 0.355 |
| (Nutr [Medium] × Stage [During]) × Sex [M] | -1.43 | -8.47 – 5.61 | 0.690 |
| (Nutr [High] × Stage [During]) × Sex [M] | -0.49 | -6.70 – 5.71 | 0.876 |
| (Nutr [Medium] × Stage [After]) × Sex [M] | 0.97 | -6.07 – 8.00 | 0.787 |
| (Nutr [High] × Stage [After]) × Sex [M] | 10.18 | 3.97 – 16.38 | **0.001** |
| (Repr [Rem] × Stage [During]) × Sex [M] | -1.96 | -8.50 – 4.58 | 0.557 |
| (Repr [Rem] × Stage [After]) × Sex [M] | 5.55 | -0.99 – 12.10 | 0.096 |
| (Nutr [Medium] × Repr [Rem] × Stage [During]) × Sex [M] | 5.93 | -3.37 – 15.22 | 0.211 |
| (Nutr [High] × Repr [Rem] × Stage [During]) × Sex [M] | -0.20 | -9.41 – 9.00 | 0.965 |
| (Nutr [Medium] × Repr [Rem] × Stage [After]) × Sex [M] | -0.62 | -9.92 – 8.67 | 0.895 |
| (Nutr [High] × Repr [Rem] × Stage [After]) × Sex [M] | -9.38 | -18.58 – -0.17 | **0.046** |
| Observations | 860 | | |
| *R*2 / *R*2 adjusted | 0.233 / 0.201 | | |
