# Supplementary material for: Environmental context shapes sex-specific costs of reproduction in a dioecious plant
Source: Ann Bot. 2025 Nov 14;137(4):1036–46. doi: 10.1093/aob/mcaf296 (PMC13095889; doi:10.1093/aob/mcaf296)
Supplement: mcaf296_Supplementary_Data [file mcaf296_supplementary_data.zip › TableS3.docx]

Table S3. Analysis of Variance (ANOVA) for the effects of nutrient treatment, reproductive investment (inflorescence removal), flowering stage, sex, and their interactions on leaf nitrogen content estimated using a SPAD meter for dioecious *Sagittaria latifolia* grown in a common garden. Values shown are the parameter estimates, their confidence intervals, and *p*-values. Fixed effects in the model were: Nutrient treatment (Nutr) with three levels: low, medium, high, reproductive investment (Repr) with two levels: intact, and removed (Rem); flowering stage with three levels: before, during, after; and sex with two levels: female and male (M).

|  | **Leaf N (SPAD units)** | | |
| --- | --- | --- | --- |
| *Predictors* | *Estimates* | *CI* | *p* |
| (Intercept) | 16.66 | 12.92 – 20.39 | **<0.001** |
| Nutr [Medium] | 5.51 | -0.19 – 11.22 | 0.058 |
| Nutr [High] | 10.14 | 5.35 – 14.92 | **<0.001** |
| Repr [Rem] | 4.97 | -0.31 – 10.25 | 0.065 |
| Stage [During] | -0.33 | -5.61 – 4.95 | 0.902 |
| Stage [After] | -2.18 | -7.46 – 3.10 | 0.418 |
| Sex [M] | -5.92 | -10.83 – -1.02 | **0.018** |
| Nutr [Medium] × Repr [Rem] | -7.51 | -15.07 – 0.05 | 0.052 |
| Nutr [High] × Repr [Rem] | -3.42 | -10.55 – 3.70 | 0.346 |
| Nutr [Medium] × Stage [During] | 2.09 | -5.98 – 10.16 | 0.611 |
| Nutr [High] × Stage [During] | 0.26 | -6.51 – 7.02 | 0.941 |
| Nutr [Medium] × Stage [After] | 0.75 | -7.32 – 8.82 | 0.856 |
| Nutr [High] × Stage [After] | 1.03 | -5.73 – 7.79 | 0.765 |
| Repr [Rem] × Stage [During] | 0.48 | -6.99 – 7.95 | 0.899 |
| Repr [Rem] × Stage [After] | -1.23 | -8.70 – 6.24 | 0.746 |
| Nutr [Medium] × Sex [M] | 4.03 | -2.97 – 11.04 | 0.259 |
| Nutr [High] × Sex [M] | 9.63 | 3.32 – 15.93 | **0.003** |
| Repr [Rem] × Sex [M] | 4.57 | -2.09 – 11.24 | 0.178 |
| Stage [During] × Sex [M] | 6.01 | -0.93 – 12.95 | 0.090 |
| Stage [After] × Sex [M] | 1.56 | -5.38 – 8.50 | 0.660 |
| (Nutr [Medium] × Repr [Rem]) × Stage [During] | -1.63 | -12.32 – 9.05 | 0.764 |
| (Nutr [High] × Repr [Rem]) × Stage [During] | -1.90 | -11.98 – 8.18 | 0.712 |
| (Nutr [Medium] × Repr [Rem]) × Stage [After] | 1.53 | -9.16 – 12.22 | 0.779 |
| (Nutr [High] × Repr [Rem]) × Stage [After] | 1.08 | -9.00 – 11.15 | 0.834 |
| (Nutr [Medium] × Repr [Rem]) × Sex [M] | -2.80 | -12.20 – 6.60 | 0.559 |
| (Nutr [High] × Repr [Rem]) × Sex [M] | -5.25 | -14.48 – 3.98 | 0.265 |
| (Nutr [Medium] × Stage [During]) × Sex [M] | -6.98 | -16.89 – 2.92 | 0.167 |
| (Nutr [High] × Stage [During]) × Sex [M] | -6.45 | -15.37 – 2.47 | 0.156 |
| (Nutr [Medium] × Stage [After]) × Sex [M] | -1.84 | -11.75 – 8.06 | 0.715 |
| (Nutr [High] × Stage [After]) × Sex [M] | -1.52 | -10.44 – 7.40 | 0.738 |
| (Repr [Rem] × Stage [During]) × Sex [M] | -6.06 | -15.49 – 3.36 | 0.207 |
| (Repr [Rem] × Stage [After]) × Sex [M] | 0.16 | -9.27 – 9.59 | 0.973 |
| (Nutr [Medium] × Repr [Rem] × Stage [During]) × Sex [M] | 6.09 | -7.20 – 19.38 | 0.369 |
| (Nutr [High] × Repr [Rem] × Stage [During]) × Sex [M] | 7.44 | -5.62 – 20.49 | 0.264 |
| (Nutr [Medium] × Repr [Rem] × Stage [After]) × Sex [M] | -0.90 | -14.19 – 12.39 | 0.894 |
| (Nutr [High] × Repr [Rem] × Stage [After]) × Sex [M] | 1.05 | -12.00 – 14.11 | 0.874 |
| Observations | 834 | | |
| *R*2 / *R*2 adjusted | 0.356 / 0.328 | | |
