# Supplementary material for: Environmental context shapes sex-specific costs of reproduction in a dioecious plant
Source: Ann Bot. 2025 Nov 14;137(4):1036–46. doi: 10.1093/aob/mcaf296 (PMC13095889; doi:10.1093/aob/mcaf296)
Supplement: mcaf296_Supplementary_Data [file mcaf296_supplementary_data.zip › TableS4.docx]

Table S4. Analysis of Variance (ANOVA) for the effects of nutrient treatment, reproductive investment (inflorescence removal), sex, and their interactions on (a) estimated leaf nitrogen content (measured in SPAD units), and (b) plant size (measures as the mid-vein length) for dioecious *Sagittaria latifolia* grown in a common greenhouse environment in the year after reproduction. Values shown are the parameter estimates, their confidence intervals, and *p*-values. Fixed effects in the model were: nutrient treatment (Nutr ) with three levels: low, medium, high, Reproductive investment (Repr) with two levels: intact, and removed (Rem); and sex with two levels: female and male (M).

| **a) Leaf nitrogen content** | | | |
| --- | --- | --- | --- |
| *Predictors* | *Estimates* | *CI* | *p* |
| (Intercept) | 14.48 | 10.57 – 18.38 | **<0.001** |
| Nutr [Medium] | 6.26 | 0.29 – 12.22 | **0.040** |
| Nutr [High] | 11.16 | 6.16 – 16.17 | **<0.001** |
| Repr [Rem] | 3.74 | -1.78 – 9.26 | 0.184 |
| Sex [M] | -4.37 | -9.50 – 0.77 | 0.095 |
| Nutr [Medium] × Repr [Rem] | -5.98 | -13.88 – 1.93 | 0.138 |
| Nutr [High] × Repr [Rem] | -2.35 | -9.80 – 5.10 | 0.536 |
| Nutr [Medium] × Sex [M] | 2.19 | -5.13 – 9.51 | 0.557 |
| Nutr [High] × Sex [M] | 8.10 | 1.51 – 14.70 | **0.016** |
| Repr [Rem] × Sex [M] | 4.73 | -2.23 – 11.70 | 0.182 |
| (Nutr [Medium] × Repr [Rem]) × Sex [M] | -3.70 | -13.52 – 6.13 | 0.459 |
| (Nutr [High] × Repr [Rem]) × Sex [M] | -4.19 | -13.84 – 5.46 | 0.393 |
| Observations | 278 | | |
| *R*2 / *R*2 adjusted | 0.371 / 0.345 | | |

| **b) Plant size** |  |  |  |
| --- | --- | --- | --- |
| *Predictors* | *Estimates* | *CI* | *p* |
| (Intercept) | 4.75 | 4.01 – 5.49 | **<0.001** |
| Nutr [Medium] | 1.27 | 0.15 – 2.40 | **0.026** |
| Nutr [High] | 2.66 | 1.71 – 3.60 | **<0.001** |
| Repr [Rem] | -0.26 | -1.30 – 0.78 | 0.628 |
| Sex [M] | -1.37 | -2.34 – -0.40 | **0.006** |
| Nutr [Medium] × Repr [Rem] | -0.41 | -1.90 – 1.08 | 0.587 |
| Nutr [High] × Repr [Rem] | 0.06 | -1.35 – 1.46 | 0.937 |
| Nutr [Medium] × Sex [M] | 0.45 | -0.93 – 1.83 | 0.520 |
| Nutr [High] × Sex [M] | -0.31 | -1.55 – 0.94 | 0.629 |
| Repr [Rem] × Sex [M] | 1.80 | 0.49 – 3.11 | **0.007** |
| (Nutr [Medium] × Repr [Rem]) × Sex [M] | -1.39 | -3.24 – 0.46 | 0.141 |
| (Nutr [High] × Repr [Rem]) × Sex [M] | -0.27 | -2.09 – 1.55 | 0.769 |
| Observations | 278 | | |
| *R*2 / *R*2 adjusted | 0.367 / 0.340 | | |
