# Supplementary figures and images for: Environmental context shapes sex-specific costs of reproduction in a dioecious plant
Source: Ann Bot. 2025 Nov 14;137(4):1036–46. doi: 10.1093/aob/mcaf296 (PMC13095889; doi:10.1093/aob/mcaf296)

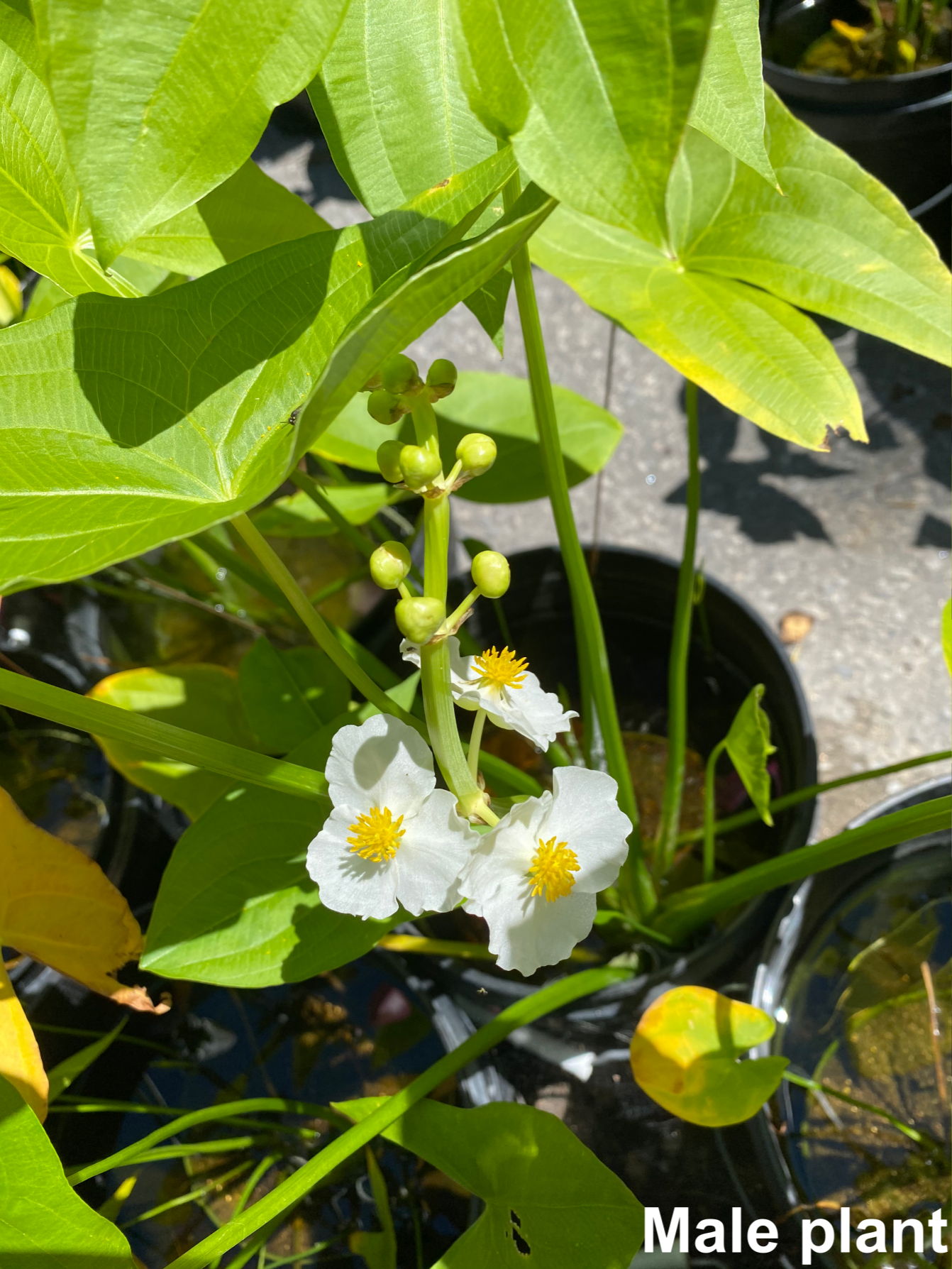

Male plant

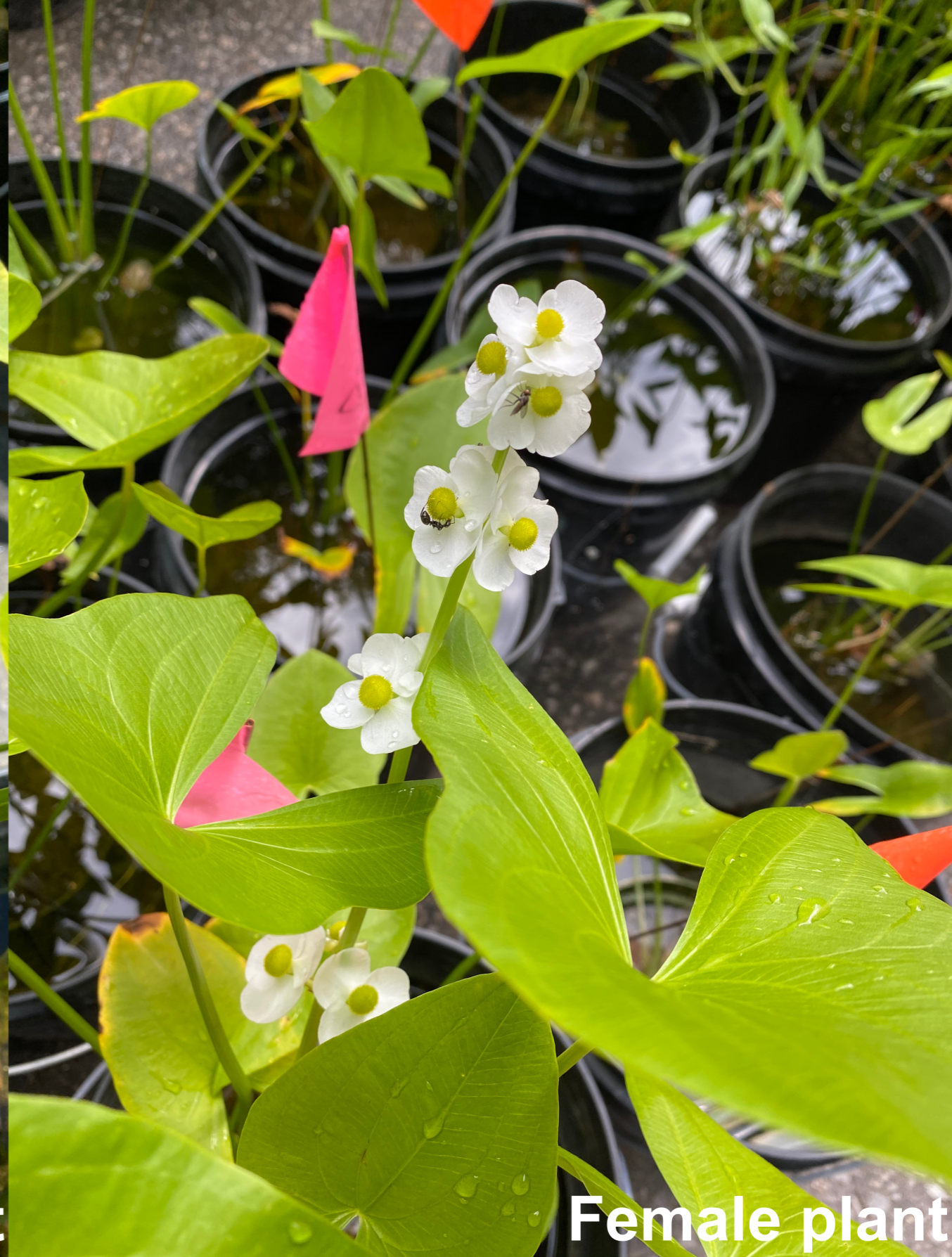

Female plant

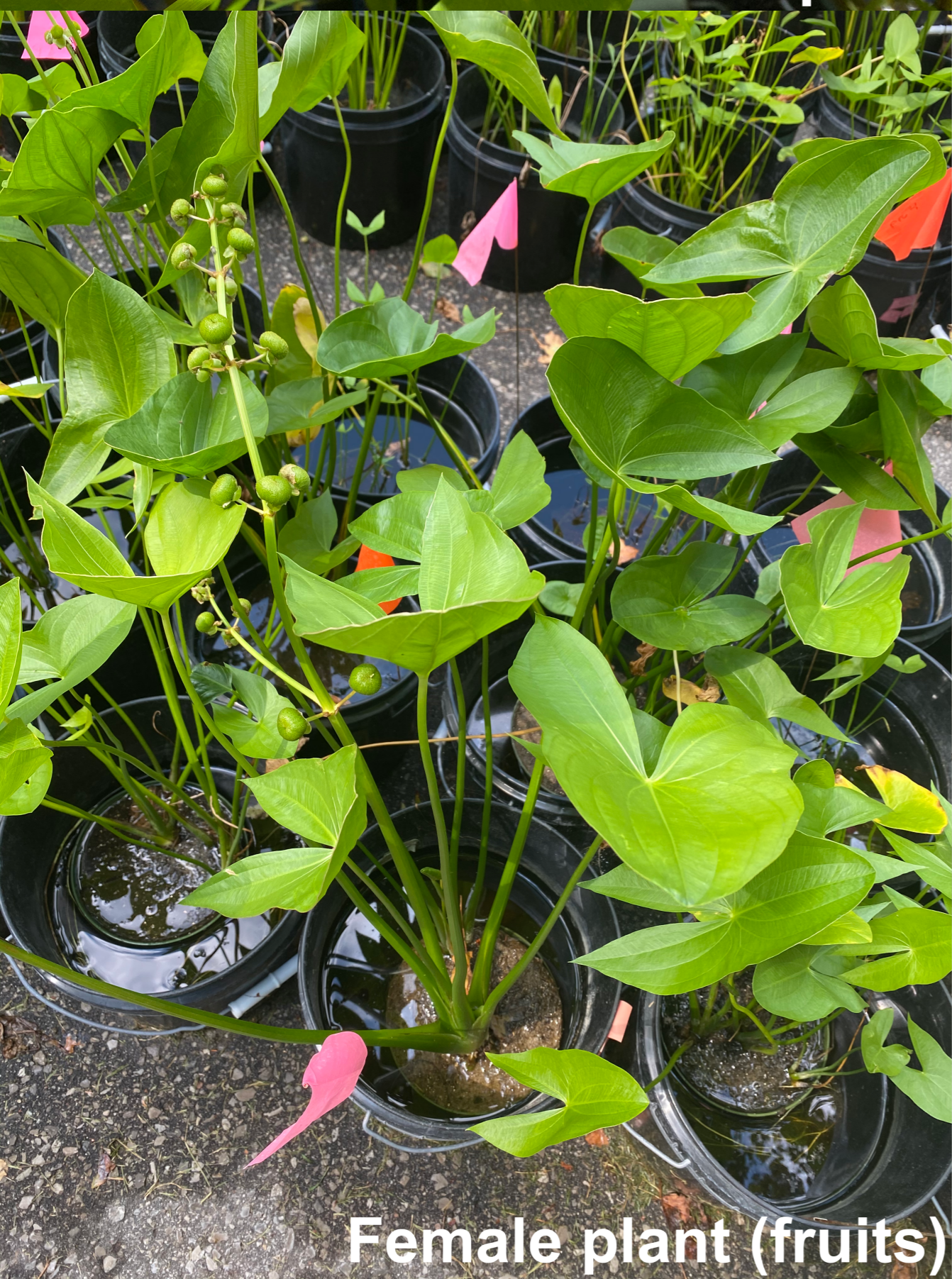

Female plant (fruits)

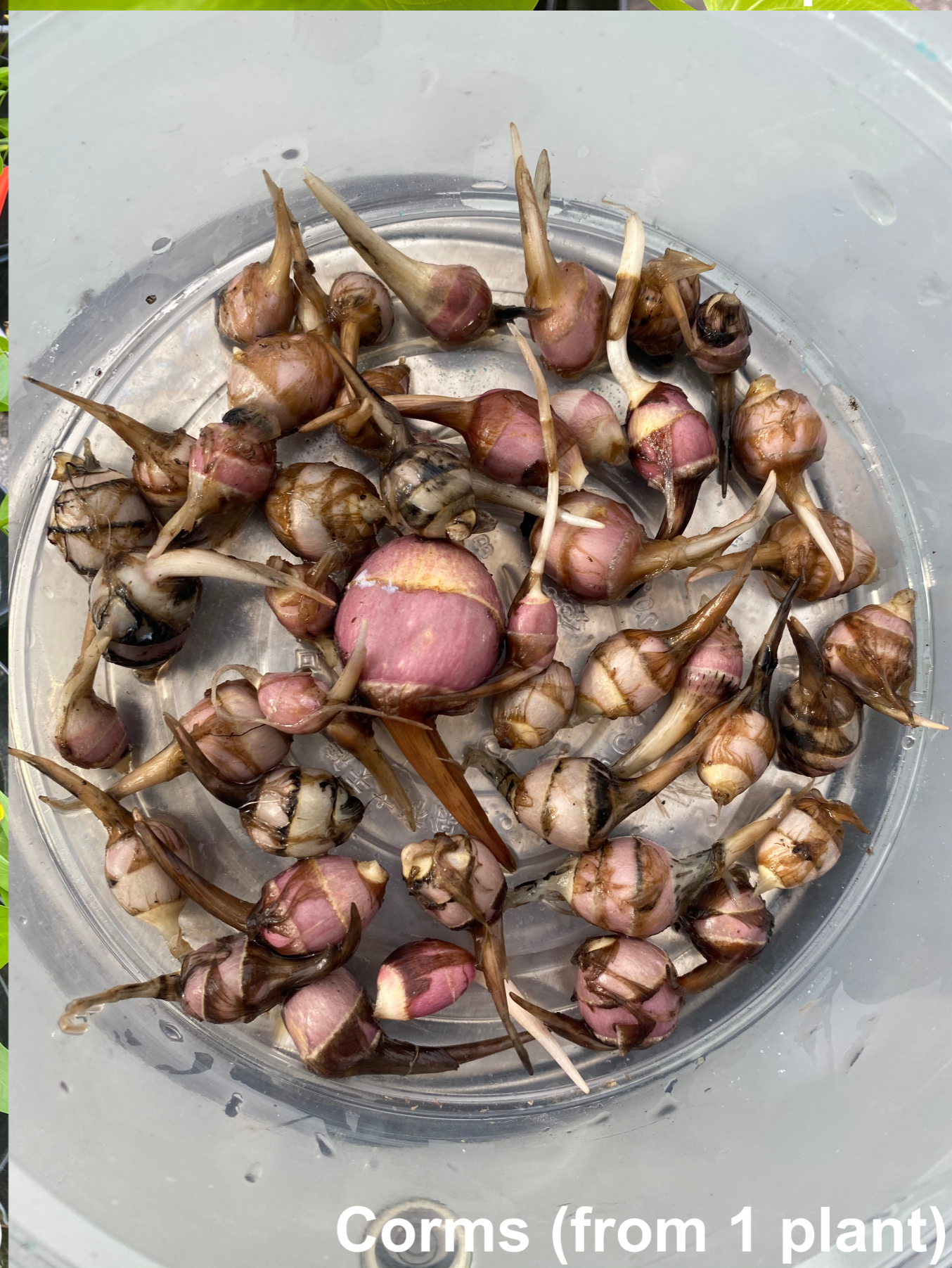

Corms (from 1 plant)

Supplement: mcaf296_Supplementary_Data [file mcaf296_supplementary_data.zip › FigS2_Sagittaria_latifolia_Flowers_Fruits_Corms.pdf]
